# Whole-Genome Sequencing of *Klebsiella pneumoniae* Isolates to Track Strain Progression in a Single Patient With Recurrent Urinary Tract Infection

**DOI:** 10.3389/fcimb.2019.00014

**Published:** 2019-02-08

**Authors:** Kristine M. Wylie, Todd N. Wylie, Patrick J. Minx, David A. Rosen

**Affiliations:** ^1^Division of Pediatric Infectious Diseases, Department of Pediatrics, Washington University School of Medicine, St. Louis, MO, United States; ^2^McDonnell Genome Institute, Washington University School of Medicine, St. Louis, MO, United States; ^3^Division of Gastroenterology, Hepatology and Nutrition, Department of Pediatrics, Washington University School of Medicine, St. Louis, MO, United States; ^4^Department of Molecular Microbiology, Washington University School of Medicine, St. Louis, MO, United States

**Keywords:** *Klebsiella pneumoniae*, whole-genome sequencing, single nucleotide variants, urinary tract infection, strain tracking

## Abstract

*Klebsiella pneumoniae* is an important uropathogen that increasingly harbors broad-spectrum antibiotic resistance determinants. Evidence suggests that some same-strain recurrences in women with frequent urinary tract infections (UTIs) may emanate from a persistent intravesicular reservoir. Our objective was to analyze *K. pneumoniae* isolates collected over weeks from multiple body sites of a single patient with recurrent UTI in order to track ordered strain progression across body sites, as has been employed across patients in outbreak settings. Whole-genome sequencing of 26 *K. pneumoniae* isolates was performed utilizing the Illumina platform. PacBio sequencing was used to create a refined reference genome of the original urinary isolate (TOP52). Sequence variation was evaluated by comparing the 26 isolate sequences to the reference genome sequence. Whole-genome sequencing of the *K. pneumoniae* isolates from six different body sites of this patient with recurrent UTI demonstrated 100% chromosomal sequence identity of the isolates, with only a small P2 plasmid deletion in a minority of isolates. No single nucleotide variants were detected. The complete absence of single-nucleotide variants from 26 *K. pneumoniae* isolates from multiple body sites collected over weeks from a patient with recurrent UTI suggests that, unlike in an outbreak situation with strains collected from numerous patients, other methods are necessary to discern strain progression within a single host over a relatively short time frame.

## Introduction

Numerous investigations have utilized whole-genome sequencing (WGS) to track the transmission of bacterial pathogens within healthcare facilities (Koser et al., 2012; Snitkin et al., 2012; Leekitcharoenphon et al., 2014). One notable example involved the 2011–2012 outbreak of a carbapenemase-producing *Klebsiella pneumoniae* strain at the National Institutes of Health Clinical Center (Snitkin et al., 2012). While epidemiologic data alone could not deduce the mode, direction, or routes of organism transmission among patients, WGS followed by single nucleotide variant (SNV) analysis was used to map the organism's movement from different anatomic sites of the index patient to the 18 patients ultimately afflicted.

In this study, we aimed to ascertain the migration of strains across body niches in a single patient with recurrent *K. pneumoniae* urinary tract infections (UTIs). While *Escherichia coli* cause the majority of UTIs, *K. pneumoniae* is the second leading etiologic agent in most populations (Flores-Mireles et al., 2015). Interestingly, more than half of recurrent *E. coli* UTIs are caused by the same strain as the original UTI (Hooton, 2001). Data suggest the original UTI strain is responsible for the majority of *K. pneumoniae* recurrences as well (Kil et al., 1997). Over the last decade, data from both humans and murine models have suggested that an intracellular reservoir of bacteria within the bladder may seed some recurrent UTIs (Mysorekar and Hultgren, 2006; Rosen et al., 2007, 2008; Liu et al., 2016). This contrasts with the traditional view of uropathogen ascension from a fecal reservoir to the periurethral niche and ultimately to the bladder (Hooton, 2001). In an attempt to distinguish these alternative routes of infection, we sequenced 26 *K. pneumoniae* isolates collected prospectively from the urine, periurethral, vaginal, fourchette, perianal, and rectal sites of a single patient.

## Materials and Methods

### Bacterial Isolates

**Figure 1:**
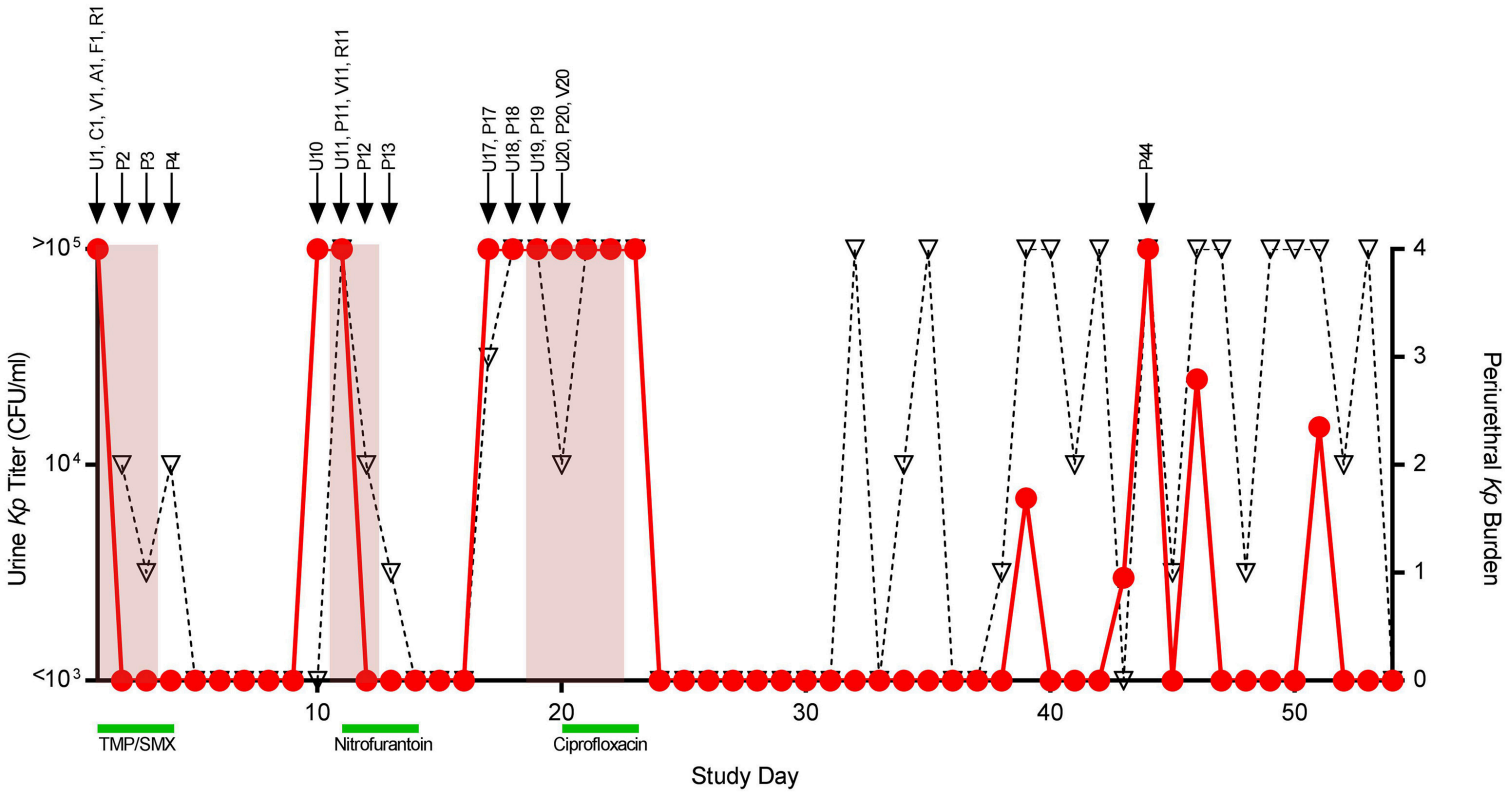
Patient course and isolates collected. Daily urine culture titers of *Klebsiella pneumoniae* (*Kp*) are shown quantified on the left y-axis (circles, red line). Daily periurethral swab titers are semi-quantitated on the right y-axis (triangles, dotted black line) with quadrant 4 indicating the highest relative burden of bacteria. Days the patient experienced urinary symptomatology are shaded in red. Antibiotic treatment courses are denoted by green bars. Arrows above the graph signify the collection date of individual strains denoted by a letter indicating collection site followed by a number indicating study day collected. Abbreviations, U, urine (self-collected, midstream); C, catheter urine; V, vaginal swab; A, perianal swab; F, fourchette swab; R, rectal swab; P, periurethral swab (self-collected); TMP-SMX, trimethoprim-sulfamethoxazole.

Summaries of the patient's clinical course and the isolates sequenced are shown in Figure 1. All bacterial isolates were obtained from one 26-year-old female with a history of recurrent UTI, as part of a larger prospective study of women with recurrent UTI at the University of Washington (Czaja et al., 2009). This study was approved by the Human Subjects Review Committee at the University of Washington, and the subject gave written informed consent in accordance with the Declaration of Helsinki. This patient's enrollment urinary *K. pneumoniae* isolate (TOP52, strain 1721; hereafter referred to as U1) has previously been sequenced and used as a model uropathogen in experimental murine UTI (Rosen et al., 2008; Johnson et al., 2014). During the study period, the patient had an initial episode of acute cystitis followed by two recurrent symptomatic episodes. Midstream urine and periurethral specimens were self-collected daily throughout the study period, and additional body sites were cultured upon clinic visits. Urine isolates were quantified from 10^3^ CFU/ml to >10^5^ CFU/ml, and periurethral isolates were scored from 1 to 4 based on growth in 4-quadrant streaking. Individual colonies were frozen in glycerol and stored at −70°C. All 26 strains of *K. pneumoniae* saved from patient TOP52 were sequenced and assigned a notation designated by a capital letter indicating body site followed by a number indicating day collected (Figure 1).

### Illumina Sequencing of Bacterial Isolates

Isolates were subcultured overnight in Luria-Bertani broth at 37°C shaking. DNA was extracted from each isolate using the Wizard Genomic DNA Prep Kit (Promega, Madison, Wisconsin). Dual-indexed Illumina sequencing libraries were constructed from each sample using the NexteraXT kit (Illumina, Inc., San Diego, California), pooled, and sequenced on the Illumina HiSeq 4000 platform.

### PacBio SMRT Sequencing of U1 Bacterial Isolate

High molecular weight DNA was extracted from the U1 isolate using the DNeasy Mericon Food Kit (Qiagen, Germantown, Maryland), with the following modifications: (a) the sample was not homogenized before beginning the protocol; (b) 150 microliters of 10 mg/mL lysozyme was added to the resuspended bacterial pellet and incubated at 37°C for 30 min with shaking at 500 rpm; (c) 50 microliters of the proteinase K solution supplied with the kit was added to the Food Lysis buffer and incubated for 90 min at 60°C with shaking at 1,000 rpm. PacBio SMRT (Single Molecule, Real-Time) sequencing libraries were constructed with target 20-kilobase insert size and sequenced on the PacBio Sequel instrument (PacBio, Menlo Park, California).

### Genome Assembly of U1 Bacterial Isolate

Our PacBio assembly for the U1 bacterial isolate was initiated using the PacBio SMRT Link analysis software suite (https://www.pacb.com/support/software-downloads/). The raw PacBio reads were assembled with HGAP4 V5.0.1.9585 (Chin et al., 2013, 2016) using default parameters, and the Arrow module was used for subsequent consensus correction.

### U1 Bacterial Isolate Genome Assembly Refinement

Illumina sequences from the same isolate used to generate our PacBio U1 assembly were aligned to the PacBio U1 assembly using BWA-MEM v0.7.17 (Li and Durbin, 2010). Subsequent Illumina read depth metrics were used to help evaluate the PacBio U1 assembly for coverage, continuity, and sequence variation (see Supplemental Table 1, TOP52-U1). Regions discordant between the Illumina and PacBio sequencing data were manually resolved using the Integrated Genomics Viewer v2.3.55 (Robinson et al., 2011; Thorvaldsdottir et al., 2013) and Consed v29.0 (Gordon and Green, 2013) to edit the assembly.

### Variant Analysis of Bacterial Isolates

Illumina sequences from all 26 bacterial isolates were mapped to our U1-derived, refined K. pneumoniae reference genome assembly using BWA-MEM v0.7.17 (Li and Durbin, 2010). Resultant SAM/BAM files underwent duplication removal (paired-end reads treated as single-end), sorting by alignment position, and indexing using SAMtools v1.7 (Li, 2011). SNVs and small indels (insertion/deletions) were called using VarScan v2.4.3 (Gordon and Green, 2013). VarScan was set to require a minimum coverage depth of 10x and minimum average quality of 30. Heterozygous calls were discarded. Indel detection in VarScan has been shown to be in the 1–30 bp size range (Koboldt et al., 2013). Additionally, Illumina read depth coverage against our reference U1 assembly was calculated for each bacterial isolate using SAMtool's depth and VarScan's readcounts sub-commands. Sequence alignments were scanned for potential “dropout,” where assembly coverage was <10x Illumina read depth, and such regions were compared across all isolates for loci concordance (see Supplemental Table 2). Potentially concordant dropout regions were then manually reviewed using Tablet (Milne et al., 2013, 2016), the graphical genome assembly viewer. For an example of commands used to call variants on U1 isolates, (see Supplemental Table 3). For metrics related to sequence coverage and variant calling, (see Supplemental Table 1). To verify that no variants distinguished the strains, the data were reanalyzed using the Snippy workflow on the Galaxy web platform using the public server at https://usegalaxy.eu/ with default parameters (Seeman, 2015; Afgan et al., 2018).

### Data Access

Sequence data are submitted to the Sequence Read Archive under BioProject Accession Number PRJNA488268. The reference genome is under BioSample Accession Number SAMN09928108. The Illumina sequence reads for each sample are under BioSample Accession Numbers SAMN10103286-SAMN10103311.

## Results

The patient had an initial episode of UTI followed by two subsequent symptomatic recurrences on days 11 and 19, each with urine cultures yielding *K. pneumoniae*. All recovered urinary isolates displayed the same antibiotic susceptibility pattern with ampicillin resistance and susceptibility to all other antimicrobials tested including the patient's treatment regimens of trimethoprim-sulfamethoxazole, nitrofurantoin, and ciprofloxacin. For each UTI, positive periurethral and urine cultures arose almost simultaneously, precluding definitive conclusions about the directionality of infection. Between study days 32 and 53, the patient had consistently high periurethral burdens of *K. pneumoniae* with only a few episodes of bacteriuria, none of which were symptomatic. A total of 26 *K. pneumoniae* isolates were saved from the patient and all underwent WGS.

The TOP52 U1 isolate was assembled using PacBio and Illumina sequence data into a single contiguous genomic sequence (5,231,007 bp), with only one ambiguous region of approximately 19 bases (pos. 2862397-2862415, see Supplemental Table 2). This refined reference improves substantially upon the previously available draft genome (GenBank acc. JNFE00000000.1), which comprised 85 discontiguous, unordered sequences (Johnson et al., 2014). Additionally, two plasmid sequences were assembled, herein P1 (171,030 bp) and P2 (65,404 bp). P1 had closest sequence identity to *K. pneumoniae* strain KpN01 plasmid (GenBank acc. CP012989.1), with 99% identity over 63% of the reference. P2 had closest sequence identity to *K. pneumoniae* strain HZW25 plasmid (GenBank acc. CP025213.1), with 99% identity over 54% of the reference. Previous capsule K-typing by the Statens Serum Institut using historical sera identified this strain as capsular type K6. However, using our TOP52 U1 genomic sequence and the Institut Pasteur *Klebsiella* Sequence Typing Database (Jolley and Maiden, 2010; Brisse et al., 2013; Institut Pasteur, 2018), we determined TOP52 U1 contains *wzi* allele 150, which corresponds to associated KL types of KL163, KL27, and KL46. The capsular carbohydrate structural differences and similarities between these KL types and K type have yet to be defined. We also determined the sequence type of this isolate to be 152.

Illumina sequences from each of the 26 isolates were aligned to the complete TOP52 U1 assembly to identify genomic variants. Genome coverage was high for each of the isolates, with the breadth of coverage ranging from 99.8152 to 99.9998% and the deduplicated depth of coverage ranging from 100.4x to 121.8x. No single-nucleotide variants or small indels (here defined as 30 bp or less) were detected in the genomes. This result was verified using an orthogonal variant calling pipeline, snippy. A conserved read dropout (avg. 0–8x) region of ~4,357 bp (pos. 59,982–64,338) was detected on plasmid P2 in 6 of the isolates collected on days 11-13 (P11, P12, P13, R11, U11, V11). The deleted region included several predicted genes encoding a chlorite dismutase (locus tag D1637_27505), a cupin domain-containing protein (locus tag D1637_27510), a hypothetical protein (locus tag D1637_27515), a SamB family protein (locus tag D1637_27520) and a thermonuclease family protein (locus tag D1637_27525). This small deletion within plasmid P2 was not found in any of the isolates collected after day 13.

## Discussion

WGS of 26 *Klebsiella pneumoniae* isolates from multiple body sites of a single patient with recurrent UTI over several weeks indicates that *K. pneumoniae* may exist clonally in some patients for extended periods of time. However, SNV and small-indel analysis was unable to specify the progression of *K. pneumoniae* strains through multiple body niches over time. Remarkably, not a single SNV was identified, demonstrating complete chromosomal identity of the isolates. If such genomic identity is generally common within individual patients, a novel approach that does not rely solely on genetic or genomic assays may be required to further specify the reservoir for recurrent UTI.

One limitation of this study is that only single colonies of *K. pneumoniae* from each body site at each time point were saved and sequenced, thus minor alternative populations of *K. pneumoniae* may have been missed. This could be important, as potentially multiple uropathogenic isolates may be carried by a woman at any given time (Chen et al., 2013). Despite this, the chromosomal sequence identity of the 26 isolates analyzed suggests that the patient was colonized by this dominant clone throughout the study period.

Upon secondary analysis, we identified a missing region of the P2 plasmid in isolates recovered from multiple sites within the patient between days 11 and 13. Though these strains likely originated from a single subclone, their synchronous appearance precludes speculation as to the niche in which this deletion arose and the order of migration of this strain across body sites. Interestingly, none of the isolates associated with the subsequent UTI exhibited this deletion, indicating they did not originate from the subclone with the P2 deletion associated with the first recurrence.

While mutation rates in *K. pneumoniae* are not definitively known, studies estimate the mutation rate to be on the order of 10^−7^ substitutions per site per year (Duchene et al., 2016). This, however, does not take into account selective pressures that may exist in different host environments. Despite the expected relatively low frequency of spontaneous mutation, SNVs identified in outbreak situations have been sufficient to effectively track environmental and inter-patient transmission (Snitkin et al., 2012). This suggests that passage of isolates through different environments over time to another host may increase the rate of mutation. The same selective pressures may not exist within a single host, and genetic drift alone may not provide sufficient SNVs over a comparatively short time period. Our data is consistent with the frequent identification of <10 SNVs between intrapatient *K. pneumoniae* isolates from gastrointestinal and extraintestinal sites over similar time frames (Gorrie et al., 2017). Another study looking at the evolution of carbapenemase-producing *K. pneumoniae* isolates from a single patient demonstrated between 3 and 38 SNV differences over months to years (Mulvey et al., 2016). WGS could potentially still be valuable in prospective, single-patient pathogen tracking if selective pressures of given niches are greatly increased above baseline, if other pathogenic species exhibit increased mutation rates within the host, or if the duration of organism collection were extended.

## Author Contributions

KW, TW, and DR conceived and designed the experiments. KW, TW, PM, and DR performed the experiments. KW, TW, and DR analyzed the data and drafted the manuscript.

### Conflict of Interest Statement

The authors declare that the research was conducted in the absence of any commercial or financial relationships that could be construed as a potential conflict of interest.

## Supplementary Material

Supplemental Table 1 Sequence coverage and variant call metrics for bacterial isolates. Illumina short reads from each specimen were aligned to our PacBio-derived TOP52 U1 assembly and associated plasmids (P1 and P2). Metrics are presented here associated with mapping rates, breadth and depth of coverage, and counts for homozygous single-nucleotide variants and small indels.

Supplemental Table 2 Review of conserved, low assembly coverage across specimens using Illumina short reads. Illumina short reads from each specimen were aligned to our PacBio-derived TOP52 U1 assembly. For each specimen, depth of coverage was reviewed at every position in our assembly, and loci with <10x coverage were compared across all other specimens. Here, conserved loci of <10x are illustrated among specimens, highlighting a region (pos. 2862397-2862415) of Illumina read coverage “dropout.”

Supplemental Table 3 Example commands for TOP52 U1 isolate. Illumina FASTQ files are aligned to our PacBio-derived TOP52 U1 assembly, mapped reads are sorted by coordinate position and indexed, duplicate alignments are marked/ignored, single nucleotide variants and small indels are called, and mapping coverage depth is calculated.

## References

[B1] Afgan E. Baker D. Batut B. Van Den Beek M. Bouvier D. Cech M.. (2018). The Galaxy platform for accessible, reproducible and collaborative biomedical analyses: 2018 update. Nucleic Acids Res. 46, w537–w544. 10.1093/nar/gky379 29790989 PMC6030816

[B2] Brisse S. Passet V. Haugaard A. B. Babosan A. Kassis-Chikhani N. Struve C.. (2013). wzi gene sequencing, a rapid method for determination of capsular type for *Klebsiella* strains. J. Clin. Microbiol. 51, 4073–4078. 10.1128/JCM.01924-13 24088853 PMC3838100

[B3] Chen S. L. Wu M. Henderson J. P. Hooton T. M. Hibbing M. E. Hultgren S. J.. (2013). Genomic diversity and fitness of *E. coli* strains recovered from the intestinal and urinary tracts of women with recurrent urinary tract infection. Sci. Transl. Med. 5:184ra160. 10.1126/scitranslmed.3005497 23658245 PMC3695744

[B4] Chin C. S. Alexander D. H. Marks P. Klammer A. A. Drake J. Heiner C.. (2013). Nonhybrid, finished microbial genome assemblies from long-read SMRT sequencing data. Nat. Methods 10, 563–569. 10.1038/nmeth.2474 23644548

[B5] Chin C. S. Peluso P. Sedlazeck F. J. Nattestad M. Concepcion G. T. Clum A.. (2016). Phased diploid genome assembly with single-molecule real-time sequencing. Nat. Methods 13, 1050–1054. 10.1038/nmeth.4035 27749838 PMC5503144

[B6] Czaja C. A. Stamm W. E. Stapleton A. E. Roberts P. L. Hawn T. R. Scholes D.. (2009). Prospective cohort study of microbial and inflammatory events immediately preceding *Escherichia coli* recurrent urinary tract infection in women. J. Infect. Dis. 200, 528–536. 10.1086/600385 19586416 PMC3674869

[B7] Duchene S. Holt K. E. Weill F. X. Le Hello S. Hawkey J. Edwards D. J.. (2016). Genome-scale rates of evolutionary change in bacteria. Microb. Genom. 2:e000094. 10.1099/mgen.0.000094 28348834 PMC5320706

[B8] Flores-Mireles A. L. Walker J. N. Caparon M. Hultgren S. J. (2015). Urinary tract infections: epidemiology, mechanisms of infection and treatment options. Nat. Rev. Microbiol. 13, 269–284. 10.1038/nrmicro3432 25853778 PMC4457377

[B9] Gordon D. Green P. (2013). Consed: a graphical editor for next-generation sequencing. Bioinformatics 29, 2936–2937. 10.1093/bioinformatics/btt515 23995391 PMC3810858

[B10] Gorrie C. L. Mirceta M. Wick R. R. Edwards D. J. Thomson N. R. Strugnell R. A.. (2017). Gastrointestinal carriage is a major reservoir of *Klebsiella pneumoniae* infection in intensive care patients. Clin. Infect. Dis. 65, 208–215. 10.1093/cid/cix270 28369261 PMC5850561

[B11] Hooton T. M. (2001). Recurrent urinary tract infection in women. Int. J. Antimicrob. Agents 17, 259–268. 10.1016/S0924-8579(00)00350-2 11295405

[B12] Institut Pasteur (2018). MLST and Whole Genome MLST Database: Klebsiella Sequence Typing (2018). Available online at: https://bigsdb.pasteur.fr/klebsiella/klebsiella.html (Accessed December 20, 2018).

[B13] Johnson J. G. Spurbeck R. R. Sandhu S. K. Matson J. S. (2014). Genome sequence of *Klebsiella pneumoniae* urinary tract isolate TOP52. Genome Announc. 2:e00668–14. 10.1128/genomeA.00668-14 24994806 PMC4082006

[B14] Jolley K. A. Maiden M. C. (2010). BIGSdb: scalable analysis of bacterial genome variation at the population level. BMC Bioinformatics 11:595. 10.1186/1471-2105-11-595 21143983 PMC3004885

[B15] Kil K. S. Darouiche R. O. Hull R. A. Mansouri M. D. Musher D. M. (1997). Identification of a *Klebsiella pneumoniae* strain associated with nosocomial urinary tract infection. J. Clin. Microbiol. 35, 2370–2374. 9276418 10.1128/jcm.35.9.2370-2374.1997 PMC229970

[B16] Koboldt D. C. Larson D. E. Wilson R. K. (2013). Using VarScan 2 for germline variant calling and somatic mutation detection. Curr. Protoc. Bioinformatics 44, 15.4.1–17. 10.1002/0471250953.bi1504s44 25553206 PMC4278659

[B17] Koser C. U. Holden M. T. Ellington M. J. Cartwright E. J. Brown N. M. Ogilvy-Stuart A. L.. (2012). Rapid whole-genome sequencing for investigation of a neonatal MRSA outbreak. N. Engl. J. Med. 366, 2267–2275. 10.1056/NEJMoa1109910 22693998 PMC3715836

[B18] Leekitcharoenphon P. Nielsen E. M. Kaas R. S. Lund O. Aarestrup F. M. (2014). Evaluation of whole genome sequencing for outbreak detection of *Salmonella enterica*. PLoS ONE 9:e87991. 10.1371/journal.pone.0087991 24505344 PMC3913712

[B19] Li H. (2011). A statistical framework for SNP calling, mutation discovery, association mapping and population genetical parameter estimation from sequencing data. Bioinformatics 27, 2987–2993. 10.1093/bioinformatics/btr509 21903627 PMC3198575

[B20] Li H. Durbin R. (2010). Fast and accurate long-read alignment with Burrows-Wheeler transform. Bioinformatics 26, 589–595. 10.1093/bioinformatics/btp698 20080505 PMC2828108

[B21] Liu S. C. Han X. M. Shi M. Pang Z. L. (2016). Persistence of uropathogenic *Escherichia coli* in the bladders of female patients with sterile urine after antibiotic therapies. J. Huazhong. Univ. Sci. Technolog. Med. Sci. 36, 710–715. 10.1007/s11596-016-1649-9 27752899

[B22] Milne I. Bayer M. Stephen G. Cardle L. Marshall D. (2016). Tablet: visualizing next-generation sequence assemblies and mappings. Methods Mol. Biol. 1374, 253–268. 10.1007/978-1-4939-3167-5_14 26519411

[B23] Milne I. Stephen G. Bayer M. Cock P. J. Pritchard L. Cardle L.. (2013). Using tablet for visual exploration of second-generation sequencing data. Brief Bioinform. 14, 193–202. 10.1093/bib/bbs012 22445902

[B24] Mulvey M. R. Haraoui L. P. Longtin Y. (2016). Multiple variants of *Klebsiella pneumoniae* producing carbapenemase in one patient. N. Engl. J. Med. 375, 2408–2410. 10.1056/NEJMc1511360 27974041

[B25] Mysorekar I. U. Hultgren S. J. (2006). Mechanisms of uropathogenic *Escherichia coli* persistence and eradication from the urinary tract. Proc. Natl. Acad. Sci. U.S.A. 103, 14170–14175. 10.1073/pnas.0602136103 16968784 PMC1564066

[B26] Robinson J. T. Thorvaldsdottir H. Winckler W. Guttman M. Lander E. S. Getz G.. (2011). Integrative genomics viewer. Nat. Biotechnol. 29, 24–26. 10.1038/nbt.1754 21221095 PMC3346182

[B27] Rosen D. A. Hooton T. M. Stamm W. E. Humphrey P. A. Hultgren S. J. (2007). Detection of intracellular bacterial communities in human urinary tract infection. PLoS Med. 4:e329. 10.1371/journal.pmed.0040329 18092884 PMC2140087

[B28] Rosen D. A. Pinkner J. S. Jones J. M. Walker J. N. Clegg S. Hultgren S. J. (2008). Utilization of an intracellular bacterial community pathway in *Klebsiella pneumoniae* urinary tract infection and the effects of FimK on type 1 pilus expression. Infect. Immun. 76, 3337–3345. 10.1128/IAI.00090-08 18411285 PMC2446714

[B29] Seeman T. (2015). Snippy: Fast Bacterial Variant Calling From NGS Reads.. Available online at: https://github.com/tseemann/snippy (Accessed September 25, 2018).

[B30] Snitkin E. S. Zelazny A. M. Thomas P. J. Stock F. Henderson D. K. Palmore T. N.. (2012). Tracking a hospital outbreak of carbapenem-resistant *Klebsiella pneumoniae* with whole-genome sequencing. Sci. Transl. Med. 4:148ra116. 10.1126/scitranslmed.3004129 22914622 PMC3521604

[B31] Thorvaldsdottir H. Robinson J. T. Mesirov J. P. (2013). Integrative Genomics Viewer (IGV): high-performance genomics data visualization and exploration. Brief Bioinform. 14, 178–192. 10.1093/bib/bbs017 22517427 PMC3603213

