# Supplementary material for: Whole-Genome Sequencing of Klebsiella pneumoniae Isolates to Track Strain Progression in a Single Patient With Recurrent Urinary Tract Infection
Source: Front Cell Infect Microbiol. 2019 Feb 8;9:14. doi: 10.3389/fcimb.2019.00014 (PMC6375827; doi:10.3389/fcimb.2019.00014)
Supplement: Supplemental Table 3 — Example commands for TOP52 U1 isolate. Illumina FASTQ files are aligned to our PacBio-derived TOP52 U1 assembly, mapped reads are sorted by coordinate position and indexed, duplicate alignments are marked/ignored, single nucleotide variants and small indels are called, and mapping coverage depth is calculated. [file Table_3.DOCX]

# Convert SAM/BAM file to R1 & R2 FASTQ.

samtools fastq -1 KPAA-TOP52-U1_gerald_HCK5HBBXX_8_AGGCAGAA-CTCTCTAT.R1.fastq -2 KPAA-TOP52-U1_gerald_HCK5HBBXX_8_AGGCAGAA-CTCTCTAT.R2.fastq KPAA-TOP52-U1_gerald_HCK5HBBXX_8_AGGCAGAA-CTCTCTAT.bam

# Map Illumina FASTQ sequences to the PacBio reference assembly using BWA-MEM.

bwa mem K_pneumoniae_PB.fasta KPAA-TOP52-U1_gerald_HCK5HBBXX_8_AGGCAGAA-CTCTCTAT.R1.fastq KPAA-TOP52-U1_gerald_HCK5HBBXX_8_AGGCAGAA-CTCTCTAT.R2.fastq > KPAA-TOP52-U1_gerald_HCK5HBBXX_8_AGGCAGAA-CTCTCTAT.bwa.sam

# No longer need intermediate files, remove them.

\rm KPAA-TOP52-U1_gerald_HCK5HBBXX_8_AGGCAGAA-CTCTCTAT.R1.fastq

\rm KPAA-TOP52-U1_gerald_HCK5HBBXX_8_AGGCAGAA-CTCTCTAT.R2.fastq

# Convert SAM to BAM using SAMtools.

samtools view -Sb KPAA-TOP52-U1_gerald_HCK5HBBXX_8_AGGCAGAA-CTCTCTAT.bwa.sam > KPAA-TOP52-U1_gerald_HCK5HBBXX_8_AGGCAGAA-CTCTCTAT.bwa.bam

# No longer need intermediate files, remove them.

\rm KPAA-TOP52-U1_gerald_HCK5HBBXX_8_AGGCAGAA-CTCTCTAT.bwa.sam

# Positional sorting of the alignments using SAMtools.

samtools sort KPAA-TOP52-U1_gerald_HCK5HBBXX_8_AGGCAGAA-CTCTCTAT.bwa.bam -o KPAA-TOP52-U1_gerald_HCK5HBBXX_8_AGGCAGAA-CTCTCTAT.bwa.srt.bam

# No longer need intermediate files, remove them.

\rm KPAA-TOP52-U1_gerald_HCK5HBBXX_8_AGGCAGAA-CTCTCTAT.bwa.bam

# Removal of duplicate sequences using SAMtools.

samtools rmdup -S KPAA-TOP52-U1_gerald_HCK5HBBXX_8_AGGCAGAA-CTCTCTAT.bwa.srt.bam KPAA-TOP52-U1_gerald_HCK5HBBXX_8_AGGCAGAA-CTCTCTAT.bwa.dedup.bam

# No longer need intermediate files, remove them.

\rm KPAA-TOP52-U1_gerald_HCK5HBBXX_8_AGGCAGAA-CTCTCTAT.bwa.srt.bam

# Index the sorted BAM file using SAMtools.

samtools index KPAA-TOP52-U1_gerald_HCK5HBBXX_8_AGGCAGAA-CTCTCTAT.bwa.dedup.bam

# Generate a mapping stats file using SAMtools.

samtools flagstat KPAA-TOP52-U1_gerald_HCK5HBBXX_8_AGGCAGAA-CTCTCTAT.bwa.dedup.bam > KPAA-TOP52-U1_gerald_HCK5HBBXX_8_AGGCAGAA-CTCTCTAT.bwa.srt.bam.flagstat

# SNV calling using VarScan2. Requirements: 10X coverage; Q30

samtools mpileup -A -f K_pneumoniae_PB.fasta KPAA-TOP52-U1_gerald_HCK5HBBXX_8_AGGCAGAA-CTCTCTAT.bwa.dedup.bam | java -jar VarScan.v2.4.3.jar mpileup2snp --min-coverage 10 --min-avg-qual 30 --output-vcf 1 > KPAA-TOP52-U1_gerald_HCK5HBBXX_8_AGGCAGAA-CTCTCTAT.bwa.srt.bam.varscan

# Coverage metrics using Varscan2. Requirements: 10X coverage; Q30

samtools mpileup -A -f K_pneumoniae_PB.fasta KPAA-TOP52-U1_gerald_HCK5HBBXX_8_AGGCAGAA-CTCTCTAT.bwa.dedup.bam | awk '($4>0)' | java -jar VarScan.v2.4.3.jar readcounts --min-coverage 10 --min-base-qual 30 --output-vcf 1 --output-file KPAA-TOP52-U1_gerald_HCK5HBBXX_8_AGGCAGAA-CTCTCTAT.bwa.srt.bam.varscan.counts

# INDEL calling using VarScan2. Requirements: 10X coverage; Q30

samtools mpileup -A -f K_pneumoniae_PB.fasta KPAA-TOP52-U1_gerald_HCK5HBBXX_8_AGGCAGAA-CTCTCTAT.bwa.dedup.bam | java -jar VarScan.v2.4.3.jar mpileup2indel --min-coverage 10 --min-avg-qual 30 --output-vcf 1 > KPAA-TOP52-U1_gerald_HCK5HBBXX_8_AGGCAGAA-CTCTCTAT.bwa.srt.bam.varscan.indels

# Coverage metrics using SAMtools. (no depth requirements)

samtools depth -a KPAA-TOP52-U1_gerald_HCK5HBBXX_8_AGGCAGAA-CTCTCTAT.bwa.dedup.bam > KPAA-TOP52-U1_gerald_HCK5HBBXX_8_AGGCAGAA-CTCTCTAT.bwa.srt.bam.samtools.depth
